# Rasch analysis of the London Handicap Scale in stroke patients: a cross-sectional study

**DOI:** 10.1186/1743-0003-11-114

**Published:** 2014-07-31

**Authors:** Eun-Young Park, Yoo-Im Choi

**Affiliations:** 1 Department of Secondary Special Education, College of Education, Jeonju University, Jeonju, Jeollabuk-do, Republic of Korea; 2 Department of Occupational Therapy, School of Medicine, Wonkwang University, PO Box 570–749, 460 Iksandae-ro, Iksan, Jeollabuk-do, Republic of Korea

**Keywords:** London Handicap Scale, Rasch analysis, Stroke

## Abstract

**Background:**

Although activity and participation are the target domains in stroke rehabilitation interventions, there is insufficient evidence available regarding the validity of participation measurement. The purpose of this study was to investigate the psychometric properties of the London Handicap Scale in community-dwelling stroke patients, using Rasch analysis.

**Methods:**

Participants were 170 community-dwelling stroke survivors. The data were analyzed using Winsteps (version 3.62) with the Rasch model to determine the unidimensionality of item fit, the distribution of item difficulty, and the reliability and suitability of the rating process for the London Handicap Scale.

**Results:**

Data of 16 participants did not fit the Rasch model and there were no misfitting items. The person separation value was 2.42, and the reliability was .85; furthermore, the rating process for the London Handicap Scale was found to be suitable for use with stroke patients.

**Conclusions:**

This was the first trial to investigate the psychometric properties of the London Handicap Scale using Rasch analysis; the results supported the suitability of this scale for use with stroke patients.

## Background

Stroke is one of the most common chronic conditions observed in aging populations. While stroke mortality rates have declined due to developments in medicine [1,2], one-third of stroke victims are left with significant permanent disability [3,4]. Stroke survivors require rehabilitation services and long-term care and support. Numerous stroke survivors will never recover their prestroke level of functioning [5].

Disability is not only a health problem but also a complex phenomenon reflecting the interaction between the features of a person’s body and the society in which they live. Participation in the community enhances the well-being of people with disabilities. It can also decrease the long-term costs of care and support. Participation measurement is critical in determining the effects of rehabilitation interventions. Participation is the main goal in rehabilitation, which is largely due to its positive benefits. Given the requirement for participation in rehabilitation, rates of participation in people with disabilities are far lower than those observed in people without disabilities [6,7].

While interventions designed to reduce motor impairment and disabilities have traditionally been the most common focus in stroke rehabilitation, the concept of participation has received much interest since the World Health Organization’s publication of the International Classification of Functioning, Disability, and Health (ICF) [8]. The ICF suggests that impairment and disability should be viewed in terms of their social aspects, and rehabilitation intervention should focus on correcting activity limitations and participation restrictions rather than motor impairments [8]. Currently, activity and participation are the target domains in rehabilitation intervention [9,10]. This paradigm shift has increased the demand for measures to assess stroke survivors’ participation.

Although participation is a multidimensional construct, and there is no gold standard for participation [11], a valid tool for participation measurement is needed. Despite the fact that more than 30 methods of participation measurement can be found in literature [12], there is no agreement with respect to which of these are appropriate tools. The main reason for disagreement is the difficulty of operation definition on participation and subjective characteristics [13]. Participation measurement is required for rehabilitation program planning, monitoring and evaluation, assessing the impact of interventions [14], and providing a more objective view of recovery [15].

Participation is one of the areas in which high levels of measurement have not been achieved and well-developed and conceptually sound instruments are still required [16]. Some measurements include items that assess participation levels [14], such as the London Handicap Scale (LHS) [17], which is widely used for measuring participation, because it is quick to administer. The LHS is well known as a means of assessing the ICF’s concept of participation restrictions [13]. The psychometric properties of the LHS, including its validity [18], reliability [17], and responsiveness [19], have been reported in previous research.

Although many studies have used the LHS to measure participation in stroke patients [20,21], few have specifically examined the psychometric properties of the LHS in this group through item response theory. The uses of tools that do not examine these psychometric properties are likely to lead to difficulties with respect to the reproduction of the results of intervention or research.

Because measurement tools with good psychometric properties are essential in documenting the effects or impacts of any clinical intervention [16], searching for a valid tool for the measurement of participation, which is one of the major outcomes of stroke rehabilitation, is essential. Many factors affect a researcher’s choice regarding the most appropriate psychological test to use for a specific application. These factors include study sample characteristics, practical issues such as respondent burden and mode of administration, the original purpose of candidate instruments, and psychometric properties [22]. It is important to evaluate the psychometric properties of a test systematically within a specific population before the test is used for that population, because these properties are affected by population characteristics [23].

The traditional method for examining psychometric properties is based on classical test theory. More recently, item response theory, which is based on the application of a related mathematical model, has become the predominant paradigm and is considered superior to classical test theory. Rasch analysis, a specific form of item response theory, is employed not only to evaluate the validity and development of outcome measures but also to examine the validity of an instrument within a clinical population [24,25]. Rasch analysis is less sample-dependent and more broadly useful relative to classical test theory, because it provides a more comprehensive understanding of the latent structure of the test [26].

The purpose of this study was to investigate the psychometric properties of the LHS in community-dwelling stroke patients using Rasch analysis in order to determine the utility of the LHS for stroke patients. Evidence regarding the psychometric properties of the LHS in stroke patents will facilitate research into participation interventions and provide valid data regarding participation in stroke patients.

## Methods

### Participants

The sample for this cross-sectional study was chosen from community-dwelling stroke patients visiting a convalescent center for people with disabilities in South Korea. Ethical approval was granted by the ethics review board at our affiliated university, and participants were assured of confidentiality and their anonymity. The only exclusion criterion for the study was cognitive dysfunction, as demonstrated by a score of ≤18 on the Korean version of the Mini Mental State Examination (MMSE-K). Interviews were carried out by trained registered physical therapists. Questionnaire responses and participation measurements for 170 participants were analyzed; none had missing data. The participants’ ages ranged from 20 to 87 years, with an average age of 55.34 years (SD = 12.23), and 21.8% of the participants were female. The stroke diagnosis periods ranged from 3 to 360 months, with an average of 51.20 months (SD = 52.60). The mean score for the MMSE-K was 23.89 (SD = 2.85).

### Measurement

The LHS was administered to all participants. The LHS is a self-administered, six-item questionnaire that assesses the impact of chronic disease in six dimensions: orientation, physical independence, mobility, occupation, social interaction, and economic self-efficiency [11]. Each dimension is rated from 1 to 6.

### Statistical analysis

Rasch analysis has been used to aid the construction and validation of health status questionnaires for various patient groups, including stroke patients [27-29]. Rasch analysis is a unidimensional model that assumes that an item response is the result of an interaction between the scale item response and the respondent’s ability. Rasch analysis is referred to as a “rating scale model” [30] and is appropriate for modeling Likert-type response data. In this study, the rating scale model was used because the LHS consisted of a Likert scale and employed the same rating scale for all items. Data were analyzed using the Winsteps program (Version 3.62) [31].

Two indices, including the infit mean square statistic (infit MNSQ) and the outfit mean square statistic (outfit MNSQ) were used to confirm unidimensionality, examine whether items contributed adequately to the LHS domains, and identify misfitting items. The infit MNSQ is a residual and is sensitive to the person’s estimated abilities, while the outfit MNSQ is sensitive to unexpected outliers for either person or item parameters [32]. In this study, if the item or participant infit fell between 0.60 and 1.40, it was considered to fit the model appropriately [33].

Items that are used to describe a construct are arranged in hierarchical order of difficulty along a continuum. In Rasch analysis, both the person’s ability and the item difficulty are expressed as logits, which are natural logarithms of the odds of a person being able to perform a specific task. Logits with a greater positive magnitude show increasing item difficulty [27]. The reliability was examined using the person separation reliability statistic. The separation index (SI) must exceed 2 to achieve the desired level of separation reliability (i.e., a value of 0.80) and exceed 3 to attain a value of 0.90 [34].

Each item was defined by a series of threshold parameters describing the difficulty or probability of the response categories in Rasch analysis. The rating scale analysis includes category frequencies, average measures, threshold estimates, probability curves, and category fit. An item’s rating scale was considered appropriate if the threshold increased by at least 1.4 logits between categories [35].

## Results

**Figure 1:**
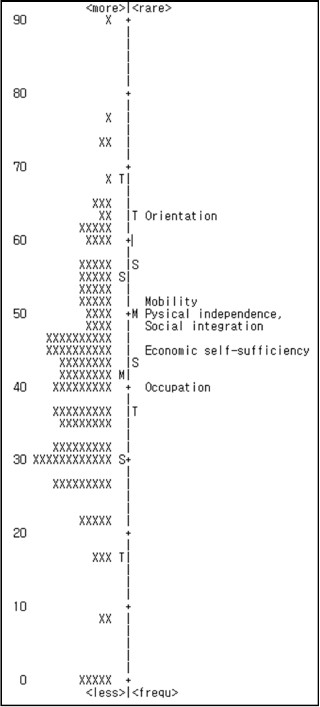
**Person ability/item difficulty map of the six LHS items.** X: one person; M: mean; S: 1 SD from the mean; T: 2 SD from the mean.

Sixteen participants did not fit the model, because their standard infit value exceeded 2.0; therefore, these participants were excluded. There were no misfitting items (Table 1). Figure 1 shows the distribution of person measures and item locations plotted along the same ability level, in addition to the hierarchical order of the six items.

**Table 1:** Item fit statistics: entry order

**Item**	**Measure**	**S.E.**	**Infit**		**Outfit**	
			**MNSQ**	**Z-value**	**MNSQ**	**Z-value**
1. Mobility	51.24	.95	.63	-3.60	.61	-3.8
2. Physical independence	49.22	.93	1.15	1.30	1.07	.60
3. Occupation	40.46	.90	.85	-1.40	.83	-1.6
4. Social integration	50.53	.94	.92	-.70	.88	-1.0
5. Orientation	63.20	1.12	1.00	.10	.95	-.30
6. Economic self-sufficiency	45.35	.91	1.38	3.00	1.50	3.8

MNSQ: Mean Square; SE: Standard Error.

The person separation was 2.42 and the reliability coefficient was 0.85. The reliability of all six items was at an acceptable level for stroke patients.

**Figure 2:**
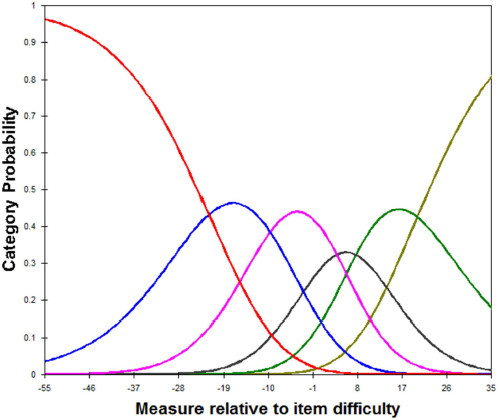
Category probability curve of the LHS.

The rating scale analysis is summarized in Table 2, and the category probability curve is depicted in Figure 2. The average modeled LHS measures for all patients who chose each response category increased with the category values. The threshold increased more than 1.4 logits between categories. Furthermore, the infit and outfit statistics appeared adjacent. Therefore, the rating scale, from 1 to 6, was determined to be suitable for use with stroke patients.

**Table 2:** Summary of the rating scale analysis

**Category label**	**Observed average**	**Infit MNSQ**	**Outfit MNSQ**	**Structure calibration**
1	-23.71	1.10	41.06	None
2	-14.67	1.01	.99	-21.95
3	-5.31	0.98	.94	-9.63
4	.54	1.10	1.06	3.82
5	9.94	0.81	.80	7.69
6	16.12	.93	.94	20.07

## Discussion

The objective of this study was to use Rasch analysis to validate the LHS for use with community-dwelling stroke patients. We investigated its unidimensionality through item fit, the distribution of item difficulty and reliability, and the suitability of the rating scale. The reliability of the LHS was satisfactory and the rating scale was suitable for use with stroke patients. Furthermore, there were no misfitting items, and the LHS showed unidimensionality.

Item fit is a tool for determining the unidimensionality of a psychometric measure, showing how each item fits in a single dimension. The fit statistics of the six items supported the proposed unidimensionality of the LHS. A high MNSQ value for an item indicates that item is not homogenous with the other items, whereas a low MNSQ value indicates that an item is a duplicate of another [36]. The ideal value of MNSQ is 1 [37]. In this study, we chose a range of 0.60–1.40 for the infit MNSQ and an outfit MNSQ of greater than 1.40 to determine whether the scale items fit the model. The economic self-sufficiency item was the closest to being a misfit, having an inordinately high MNSQ value. The possibility of different dimensions of economic self-sufficiency was reported in a previous study investigating the validity of the LHS [38]. An exploratory factor analysis to confirm the construct validity of the LHS presented two factors. One factor includes all items, with the exception of economic self-sufficiency. Although the fit indices of economic self-sufficiency were acceptable, further investigation of this phenomenon is required.

Various validity values for the LHS in stroke patients have been reported in both chronic and acute phases via classical test theory. Harwood et al. [17] reported correlation coefficients of 0.56 with the Barthel Index, 0.69 with the Nottingham Extended Activities for Daily Living Scale total scores, -0.42 with Nottingham Health Profile total scores, and -0.42 with the Geriatric Depression Scale in chronic stroke patients. Hershkovitz et al. [39] provided evidence of the validity of the LHS in acute stroke patients with correlation coefficients of -0.52 with the FIM, -0.46 with the Nottingham Prognostic Index, and 0.35 with the Timed Get Up and Go test. Reasonable face validity was also reported [18]. The validity found in previous studies based on classical test theory, and the construct validity found via item response theory in this study provides evidence for the psychometric properties of the LHS.

Person separation in the Rasch model is equivalent to Cronbach’s α [31]. In this study, the person separation value indicates how well the measure differentiates participants on the basis of their participation [27]. The minimum recommended acceptable level of person separation is .80 [40]. The present study showed a person separation value of .85. This value was consistent with Westergren and Hagell’s (2006) study [41] reporting a Cronbach’s α of .85, which was higher than that of .80 found by Chau (2009) [42]. Jenkinson et al. [10] reported internal consistency of 0.83 in acute stroke patients. Test-retest reliability has been found in previous studies; for example, Harwood et al. [17] reported a value of 0.91 in 89 chronic stroke patients.

Item difficulty was analyzed by comparing person ability and item difficulty. When person ability is consistent with item difficulty—that is, the distribution ranges of the individual attribute and item difficulty are similar—this indicates that the item difficulty is adequate [36]. Approximately 40% of participants exhibited ability scores that were markedly lower than those of the related item difficulty. This indicates that the item difficulty of the LHS was slightly high for stroke patients. The easiest item was occupation, while the most difficult was orientation. The LHS rating scale was suitable for use with stroke patients. In other words, the category measures for all items increased in the same direction.

The LHS provides an overall handicap severity score, which can be calculated with traditional weighted scoring or simple unweighted scoring. Overall handicap severity scores range from 0 to 1. Scale values of 1 and 0 indicate normal function and total disability, respectively. Each item has a weighted value; for example, the value of no mobility disadvantage is 0.071. The overall handicap severity score is calculated by summing all six utility values plus 0.456 [43]. The simple unweighted scoring system, which was suggested by Jenkinson et al. [10], rates disability from 0 (extreme disability) to 5 (no disability) for each of the six items, sums the scores, and multiplies the total by (100/30). Simple summation scoring was recommended for the LHS, because it is easier to calculate and interpret [10]. In addition, the unweighted scoring system has shown similar results as those observed with traditional weighted scoring [10] in stroke patients.

This study had some limitations. Because community-dwelling stroke patients visiting a convalescent center for people with disabilities in South Korea participated in this study, the results were only applicable to these participants. Further studies should be conducted to investigate unidimensionality. The wide age range could have been strength or a weakness. Previous studies have involved older patients than those included in this study. In two such studies, Hershovitz et al. [39] reported mean ages of 71 and 69 years for men and women, respectively, and Jenkinson et al. [10] reported a mean age of 74 for their entire sample. As a result of the wide age range of 20–87 years, the results of this study could be applicable to a wide range of patients; however, future studies should confirm whether the psychometric properties differ according to age range. Another limitation was that the participants in this study were chronic stroke patients. The unidimensionality of the LHS should be examined in acute stroke patients to allow for comprehensive and effective use of the scale. The responsiveness of measurement is a major focus in rehabilitation clinics and research. LHS scores at discharge had changed significantly with respect to mobility, physical independence, and occupation in study that assessed the effects of a day rehabilitation program on handicap in stroke patents [39]. Harwood and Ebrahim [44] reported that the LHS was reasonably responsive in hip replacement patients. In acute stroke patients, the LHS has been found to be more sensitive than the Barthel Index in measuring outpatient outcomes [44]. The psychometric properties of the LHS should be examined in acute stroke patients to determine the usefulness of the scale in measuring the effects of rehabilitation programs.

Further research into the dimensionality of participation is recommended to advance current knowledge regarding the most suitable means of measuring this construct. Item response theory enables researchers to confirm the assumption of a hierarchy of item difficulty, which is difficult to achieve using objective participation measures [12] and measurement dimensions. The validation of the participation measure in stroke survivors forms the basis of activity participation as a primary rehabilitation outcome in this population. This was the first study to investigate the psychometric properties of the LHS using Rasch analysis. The results of this study support the utility of the LHS for stroke patients.

## Conclusions

The purpose of this study was to examine the psychometric properties the LHS in community-dwelling stroke patients. All of the LHS items fit the Rasch model. This study demonstrated good reliability and validity for the LHS with respect to measuring participation in community-dwelling stroke patients. These findings confirmed the utility of LHS outcome measures in stroke patients.

## Abbreviations

LHS: London handicap scale; MNSQ: Mean square statistic; SI: Separation index.

## Competing interests

The authors confirm that there are no conflicts of interest with respect to this study.

## Authors’ contributions

E-YP made a substantial contribution to the interpretation and analysis of data and drafting the manuscript. Y-IC made a substantial contribution to data collection and manuscript revision. All of the authors read and approved the final manuscript.
